# Generating evidence to inform health technology assessment of treatments for SLE: a systematic review of decision-analytic model-based economic evaluations

**DOI:** 10.1136/lupus-2019-000350

**Published:** 2020-07-28

**Authors:** Sean Gavan, Ian Bruce, Katherine Payne

**Affiliations:** 1 Manchester Centre for Health Economics, Division of Population Health, Health Services Research and Primary Care, School of Health Sciences, Faculty of Biology, Medicine and Health, The University of Manchester, Manchester, UK; 2 Centre for Epidemiology Versus Arthritis, Centre for Musculoskeletal Research, Division of Musculoskeletal and Dermatological Sciences, School of Biological Sciences, Faculty of Biology, Medicine and Health, The University of Manchester, Manchester, UK; 3 NIHR Manchester Biomedical Research Centre, Manchester University NHS Foundation Trust, Manchester Academic Health Science Centre, Manchester, UK

**Keywords:** systemic lupus erythematosus, economic evaluations/burden of disease, systematic review, decision-analytic model

## Abstract

This study aimed to understand and appraise the approaches taken to handle the complexities of a multisystem disease in published decision-analytic model-based economic evaluations of treatments for SLE. A systematic review was conducted to identify all published model-based economic evaluations of treatments for SLE. Treatments that were considered for inclusion comprised antimalarial agents, immunosuppressive therapies, and biologics including rituximab and belimumab. Medline and Embase were searched electronically from inception until September 2018. Titles and abstracts were screened against the inclusion criteria by two reviewers; agreement between reviewers was calculated according to Cohen’s κ. Predefined data extraction tables were used to extract the key features, structural assumptions and data sources of input parameters from each economic evaluation. The completeness of reporting for the methods of each economic evaluation was appraised according to the Consolidated Health Economic Evaluation Reporting Standards (CHEERS) statement. Six decision-analytic model-based economic evaluations were identified. The studies included azathioprine (n=4), mycophenolate mofetil (n=3), cyclophosphamide (n=2) and belimumab (n=1) as relevant comparator treatments; no economic evaluation estimated the relative cost-effectiveness of rituximab. Six items of the CHEERS statement were reported incompletely across the sample: target population, choice of comparators, measurement and valuation of preference-based outcomes, estimation of resource use and costs, choice of model, and the characterisation of heterogeneity. Complexity in the diagnosis, management and progression of disease can make decision-analytic model-based economic evaluations of treatments for SLE a challenge to undertake. The findings from this study can be used to improve the relevance of model-based economic evaluations in SLE and as an agenda for research to inform future health technology assessment and decision-making.

## Introduction

SLE is a complex autoimmune disease that can affect many systems of the body and is characterised by an extremely heterogeneous presentation of symptoms.^1^ The estimated prevalence of SLE is low, relative to other autoimmune diseases, and variable between countries.^2 3^ People with SLE experience an uncertain trajectory of health outcomes that may comprise photosensitive skin rashes, fatigue, anaemia, neuropsychiatric manifestations and involvement of the pulmonary, cardiac and renal organ systems. Lupus nephritis occurs in up to 60% of people with SLE and may lead to end-stage renal disease.^4^ SLE is associated with higher mortality and lower quality of life compared with the general population.^5^

SLE is managed by rheumatologists and other specialists including nephrologists and dermatologists. The objective(s) of treatment are to reduce disease activity and prevent irreversible organ damage.^6^ The presentation of multifaceted symptoms and the imperfect criteria for diagnosis may increase the time to confirm a case of SLE in routine clinical practice. The development of treatments for SLE has been characterised by a landscape of clinical trials that failed to reach their primary end point due to, for example, the inclusion of patients without active disease, the influence of background concomitant therapies and the use of instruments that were insensitive to detect response to treatment.^7–9^ As a result, approved therapeutic alternatives for SLE are limited, encompassing off-label and licenced agents, and their use is subject to regional variation.^10 11^ Treatments comprise glucocorticoids to control inflammation in the short term, antimalarial agents such as hydroxychloroquine,^12^ immunosuppressive therapies such as azathioprine, methotrexate, mycophenolate mofetil, and newer biologic agents such as intravenous rituximab and belimumab.^13^

New therapeutic targets are expected to be identified and, in turn, therapies are likely to be developed in the future.^14^ Existing treatments may also be repositioned within the therapeutic paradigm and precision medicine initiatives, such as the 'MAximizing Sle ThERapeutic PotentiaL by Application of Novel and Stratified approaches' (MASTERPLANS) consortium, are aiming to identify biomarkers predictive of remission and low disease activity from treatment.^15 16^ Biomarkers may also inform an earlier diagnosis of SLE,^17^ monitoring of disease activity, and be used as inclusion criteria in randomised controlled trials (RCTs).^18 19^ Changes to the management of SLE will have a subsequent impact on cost and health outcomes that must be considered before being recommended in routine clinical practice. The use of economic evaluation will be essential to demonstrate that these new management strategies are a relatively cost-effective use of limited resources for health care and to inform the value of further research. Decision-analytic models are one method used internationally by health technology assessment agencies and decision-makers to estimate the relative cost-effectiveness of specific management strategies by synthesising all relevant evidence from different sources.^20^

Complexity in the (1) Diagnosis, (2) Management, and (3) Progression of disease can pose challenges when designing de novo decision-analytic model-based cost-effectiveness analyses for SLE. For example, structural assumptions with respect to the inclusion or exclusion of disease states and events, and the characterisation of care pathways in the presence of variation within and between decision-making jurisdictions, can have an impact on estimates of relative cost-effectiveness.^21^ The estimation of clinical input parameters, health-related quality of life and resource utilisation may also be difficult if the evidence that underpins these values are limited or of low quality. The strengths and limitations of existing economic evaluations can be useful to inform the development of a de novo economic evaluation, the availability of relevant data and an agenda for further empirical research.^22^ Therefore, the aim of this study was to understand and appraise the approaches taken to handle the complexities of a multisystem disease in published decision-analytic model-based economic evaluations of treatments for SLE.

## Method

A systematic review of published economic evaluations in SLE was conducted according to the Preferred Reporting Items for Systematic Reviews and Meta-analyses (PRISMA).^23^ The protocol for the systematic review is available from the authors on request. The criteria for inclusion in the systematic review, based on the Population, Intervention, Comparator, Outcome, Study-design framework, are reported in table 1. A full economic evaluation was defined as, ‘the comparative analysis of alternative courses of action in terms of both their costs and consequences’,^20^ which encompassed cost-effectiveness, cost-utility and cost-benefit analyses that used a decision-analytic model. Conference abstracts and manuscripts written in a non-English language were excluded.

**Table 1:** Systematic review inclusion criteria

Criteria	Definition
Population	Adults with SLE, lupus nephritis or ‘lupus’
Intervention	Any treatment
Comparator	Any treatment
Outcome	Expected costs and expected health outcomes
Study	Full economic evaluation (cost-effectiveness analysis; cost-benefit analysis; cost-utility analysis) that used a decision-analytic model

### Study identification

The Medline and Embase databases were searched electronically from inception until September 2018. The search strategy (reported in online supplementary appendix 1) comprised disease-specific terms for SLE and terms to identify published economic evaluations according to the filters reported by the Centre for Reviews and Dissemination.^24^ The title and abstract of each study identified by the search strategy were screened independently against the inclusion criteria by two authors (SG and KP). Disagreement between the authors was resolved by including the study at the abstract-review stage and making a subsequent decision about whether to include the study at the full-text review stage. The degree of agreement between reviewers was calculated according to Cohen’s κ.^25^ Studies that remained after screening were read in full by one author (SG) to determine whether the inclusion criteria were met.

10.1136/lupus-2019-000350.supp1 Supplementary data

### Data extraction and analysis

The following study features, structural decisions and parameter data were extracted and tabulated from each economic evaluation: target population; alternatives compared; country; type of economic evaluation; type of decision-analytic model; time horizon; measure of benefits; costs included; discount rate; currency; sources of data; deterministic and probabilistic sensitivity analyses; value of information (VOI) analyses; base-case results; probabilistic results; VOI results; and key drivers of relative cost-effectiveness. The Consolidated Health Economic Evaluation Reporting Standards (CHEERS) statement^26^ was used to evaluate whether each economic evaluation had reported 17 items with respect to its methods (1) In full, (2) Partially, or (3) Not at all. Concordance with the CHEERS statement was presented visually^27^ to illustrate common methodological characteristics that may have been challenging to report. Key features of the sample and specific reporting issues identified by the CHEERS statement were summarised by a narrative synthesis.

## Results

**Figure 1:**
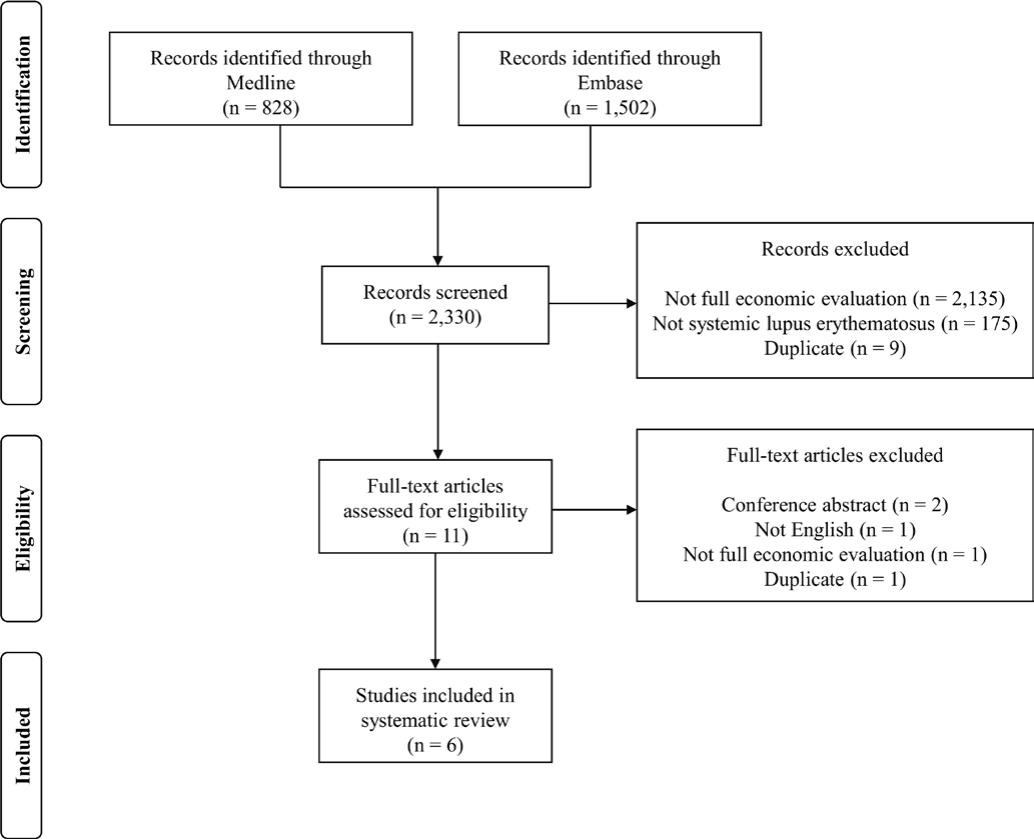
Flow diagram of included studies.

Six decision-analytic model-based economic evaluations of treatments for SLE were included in the systematic review.^28–33^ A PRISMA flow diagram of the study inclusion process is illustrated in figure 1. The key features extracted from the six economic evaluations are reported in table 2; complete data extraction tables are reported in online supplementary appendix 2. Cohen’s kappa was 0.973 which indicated almost perfect agreement between the two reviewers that screened abstracts. Eleven full-text articles were assessed against the inclusion criteria. Five full-text articles were excluded due to being conference abstracts (n=2), written in a non-English language (n=1),^34^ not being a full economic evaluation (n=1) and duplicating (reporting the same method and results) a study included in the review that was published earlier (n=1).^35^

**Table 2:** Key features of the six decision-analytic model-based economic evaluations in SLE

Study	Country	Target population	Type of model	Perspective	Type of study	Comparators	Result	VOI
Marra *et al*^28^	Canada	Patients with rheumatological conditions (predominantly SLE and RA)	Decision tree	Third-party payer	CEA	Two strategies; full-dose AZA and a genotype test to inform dose of AZA	Genotype testing strategy was dominant	No
Mohara *et al*^29^	Thailand	Patients, aged 40 years, newly diagnosed with active, severe lupus nephritis and receiving immunosuppressive therapy	Markov model	Societal	CUA	Four strategies; different combinations of IV-CYC, MMF, AZA and induction and maintenance therapies.	IV-CYC induction and AZA maintenance was dominant	No
Nee *et al*^30^	USA	Patients with lupus nephritis, between 20 years and 40 years, who responded to induction therapy	Markov microsimulation model	Societal	CUA	Two strategies; AZA and MMF	MMF had ICER of $6454 per QALY gained relative to AZA	Population EVPI: $2 058 206
Oh *et al*^31^	Korea	Adults with moderate to severe RA or SLE	Decision tree	Societal	CEA	Two strategies; weight-based dose of AZA and a genotype test to inform dose of AZA	Genotype testing strategy was dominant	No
Specchia *et al*^32^	Italy	50 000 patients with SLE that had active disease and a positive autoantibody test	Individual-level microsimulation	Italian health service and societal	CEA; CUA	Two strategies; BEL with and without SOC	BEL and SOC had ICER of €32 859 per QALY gained	No
Wilson *et al*^33^	UK	10 000 patients with lupus nephritis eligible for induction therapy	Patient-level simulation	National Health Service	CUA	Two strategies; MMF with PRED and IV-CYC with PRED	MMF with PRED was dominant	No

AZA, azathioprine; BEL, belimumab; CEA, cost-effectiveness analysis; CUA, cost-utility analysis; EVPI, expected value of perfect information; ICER, incremental cost-effectiveness ratio; IV-CYC, intravenous cyclophosphamide; MMF, mycophenolate mofetil; PRED, prednisolone; QALY, quality-adjusted life year; RA, rheumatoid arthritis; SOC, standard of care; VOI, value of information.

The economic evaluations considered azathioprine (n=4), mycophenolate mofetil (n=3), cyclophosphamide (n=2) and belimumab (n=1) as relevant comparator therapies. No published economic evaluation assessed the relative cost-effectiveness of rituximab for SLE. The types of decision-analytic model used by the studies comprised individual patient-level simulations (n=3),^30 32 33^ a cohort Markov model (n=1)^29^ and decision trees (n=2).^28 31^

**Figure 2:**
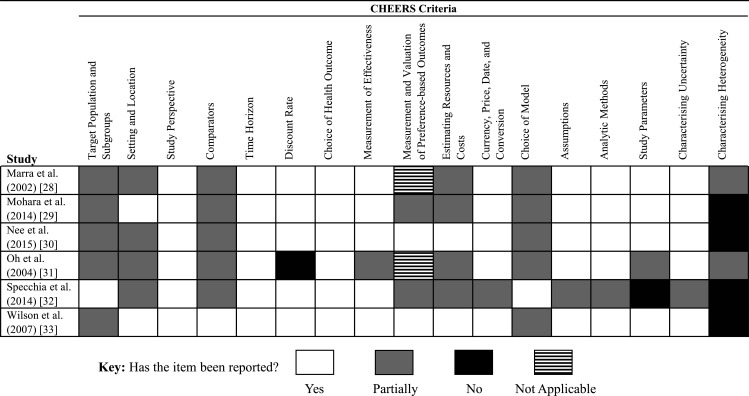
Reporting of each economic evaluation according to the Consolidated Health Economic Evaluation Reporting Standards (CHEERS) criteria.

The completeness of reporting in each manuscript, by 17 items in the CHEERS statement, is illustrated in figure 2. Six features of the economic evaluations included in the systematic review were reported incompletely across the sample (target population, comparators, measurement and valuation of preference-based outcomes, estimation of resource use and costs, choice of model, and the characterisation of heterogeneity). These six features are now described in detail.

### Target population

The target population of two economic evaluations comprised people with rheumatoid arthritis or SLE; the proportion of people with SLE or the severity of disease were not reported by either of these studies.^28 31^ Three economic evaluations specified a target population of people with lupus nephritis; however, the clinical outcome used to define the specific features of these target populations were not reported.^29 30 33^ For example, Mohara *et al*^29^ did not define active and severe lupus nephritis. Wilson *et al*^33^ did not use a clinical outcome measure to define a renal flare. Nee *et al*^30^ did not define the criteria for response to lupus nephritis induction therapy. One economic evaluation defined the target population clearly in terms of an autoantibody biomarker assessment.^32^

### Relevant comparators

All economic evaluations included in the systematic review reported justification for the intervention and immediate comparator. However, all relevant comparator therapies may not have been included by each study. For example, Nee *et al*^30^ compared azathioprine and mycophenolate mofetil for lupus nephritis maintenance therapy. By contrast, Mohara *et al*^29^ also included cyclophosphamide and compared all maintenance therapy strategies in a fully incremental analysis. Specchia *et al*^32^ compared belimumab plus ‘standard of care’ (SOC) with ‘SOC’ only. The therapies included within the ‘SOC’ arm were defined in the manuscript; however, the relative proportion of patients that received each therapy was not reported. Oh *et al*^31^ and Marra *et al*^28^ compared a genotype testing strategy with no testing before azathioprine was prescribed. The clinical context or severity of SLE was not reported by these two studies and, hence, other therapies (eg, mycophenolate mofetil) could have been included as potentially relevant comparators.

### Choice of model

Two economic evaluations structured their decision-analytic models as decision trees with a time horizon of 1 year.^28 31^ These decision trees did not characterise the progression of SLE over time. The structure of two decision-analytic models used mutually exclusive Markov health states to represent relapsing and remitting lupus nephritis over time.^29 30^ However, these Markov health states were not defined explicitly by outcome measures used in routine clinical practice. Wilson *et al*^33^ assumed a specific dose for cyclophosphamide given a lack of dose standardisation in the literature. The authors expressed that their findings may not hold if there is substantial regional variation in clinical practice.

### Health-related quality of life

Four economic evaluations reported expected health outcomes using quality-adjusted life years.^29 30 32 33^ One study^29^ estimated health-related quality of life directly from a sample of patients (n=18) in four hospitals. The three remaining studies identified health-related quality of life values from the published literature. Wilson *et al*^33^ did not identify published values for health states that characterised lupus nephritis specifically. The estimates of health-related quality of life associated with active disease and partial response to treatment were, instead, assumed to be equivalent to the values derived from having a minor and major infection, respectively. Specchia *et al*^32^ reported using published data from the Belimumab in Subjects with SLE (BLISS) trials^36 37^ to identify health-related quality of life for each health state. However, the specific health states, input parameter values or instrument of measurement were not reported in the manuscript. Nee *et al*^30^ reported that different methods (time trade-off and patient-level visual analogue scales) were used to estimate the health-related quality of life values identified within the literature.

### Resources

Specchia *et al*^32^ stated that direct health care costs (for treatments, diagnostic tests, specialist visits) were included per clinical outcome (Safety of Estrogens in Lupus Erythematosus National Assessment–Systemic Lupus Erythematosus Disease Activity Index; SELENA-SLEDAI) score and for organ damage. However, the magnitude of these costs was not reported in the manuscript. Mohara *et al*^29^ estimated health care resource use by reviewing the medical records of patients with lupus nephritis in four hospitals. The methods used to undertake this review of medical records were not reported. Nee *et al*^30^ sourced estimates of mean resource use for each Markov health state from the published literature. The authors, however, expressed uncertainty in these values by assuming they ranged above and below the mean by 25%. Oh *et al*^31^ estimated the cost of hospitalisations from four patient cases but the method to estimate these values was not reported. Marra *et al*^28^ estimated the probability and duration of hospitalisations from experts. The specific method used to elicit this information from experts was not reported in the manuscript.

### Heterogeneity

The two studies that used a decision tree evaluated a genetic testing strategy to reveal heterogeneity in response to azathioprine.^28 31^ Specchia *et al*^32^ presented an analysis for a target population that had a specific result from a biomarker test. Heterogeneity in the estimates of relative cost-effectiveness, or subgroup analyses based on patient-level characteristics, were not reported by any economic evaluation included in the systematic review.

## Discussion

This study reported a systematic review of all published decision-analytic model-based economic evaluations of treatments for SLE. Six full economic evaluations that used a decision-analytic model were identified.^28–33^ Six domains of the CHEERS criteria were reported incompletely across these economic evaluations which may indicate common challenges when producing economic evidence of treatments for SLE to decision-makers responsible for population-level resource allocation for health care: definition of the target population, choice of relevant comparators, measurement and valuation of preference-based outcomes, estimation of resource use and costs, choice and structure of the decision-analytic model, and the characterisation of heterogeneity.

Model-based economic evaluations of treatments for chronic conditions should be designed to represent the progression of disease over the lifetime of a patient cohort to account for all relevant cost and health outcomes. SLE is a complex multisystem disease; for example, patients may experience different trajectories of damage to different organ systems over time. This complexity may make the characterisation of lifetime outcomes a challenge within decision-analytic models that simulate a homogeneous cohort.^38^ The simulation of patients individually can be used to handle first-order uncertainty over the progression of lifetime outcomes.^39^ However, patient-level simulations that have complex structures may require more data to populate compared with cohort simulations which can be a barrier to their use in practice.^39^ Two economic evaluations in this systematic review, including the most recent study, simulated patients individually over a lifetime time horizon.^30 32^ By contrast, the two earliest studies conducted their analysis using decision trees.^28 31^ The use of different methods by the economic evaluations in this review may reflect the development of new analytical techniques that were available to researchers over time. Statistical analyses of the natural history of disease can underpin a decision-analytic model to characterise long-term outcomes of individual patients. For example, Watson *et al*^40^ developed a disease model for SLE using data from the Hopkins Lupus Cohort that estimated the annual change in disease activity, dose of steroids, damage to specific organ systems and mortality. This disease model was used subsequently to estimate input parameter values for the decision-analytic model by Specchia *et al*.^32^ However, results based on the Hopkins Lupus Cohort may not generalise to a wider population of patients with SLE because the cohort is characterised by a large number of African-American individuals and individuals with lower socioeconomic backgrounds.^40^ In recognition of this potential lack of generalisability, Watson *et al*^40^ suggested that further research to externally validate their disease model may be required.

The target population of the economic evaluations in this systematic review were not reported completely which limits the ability of decision-makers to determine whether the findings have relevance to their own health care jurisdictions. Inclusion criteria for clinical trials are not used as diagnostic criteria in clinical practice and, therefore, are not likely to be appropriate to define target populations of cost-effectiveness analyses.^41^ Defining target populations using (1) Clinical outcomes (eg, a score to indicate disease activity or damage), (2) Positioning on care pathways (eg, after inadequate response to hydroxychloroquine), or (3) Molecular biomarkers, can improve the relevance of economic evaluations for SLE with respect to the requirements of decision-makers. In general, the reporting of cost-effectiveness analyses has improved as guidelines for reporting have been adopted by publishers; this trend may help to explain why the most recent study in this systematic review defined their target population explicitly.

There are few RCTs of the existing treatments currently used to manage SLE and, as a consequence, treatment decisions often rely on using medicines outside of their licensed indication. The lack of an economic evaluation of rituximab, specifically, and of other treatments for SLE, more generally, may be due to a paucity of RCT evidence, relevant to patients with SLE, on which to base economic analyses. Off-label prescribing decisions incur opportunity costs and health benefits which should be evaluated to understand their relative cost-effectiveness. Rituximab, for example, was approved for managing SLE in National Health Service England via an interim clinical commissioning policy as part of the specialised services scheme which prioritises treatments for complex or rare conditions according to their cost and evidence of clinical benefit.^42^ Evidence of clinical benefit in this commissioning policy referred to open-label studies and secondary analyses of RCTs.^42^ No economic evaluation identified by this systematic review included rituximab in a fully incremental analysis as either an intervention or comparator strategy to provide evidential support for this commissioning policy. Future research to estimate the relative cost-effectiveness of rituximab for SLE may, therefore, provide useful information for decision-makers in different health care systems internationally.

The structure of a decision-analytic model should reflect the current and future pathways of care within a health care system.^43^ It may be challenging for decision-makers to determine whether the results of an economic evaluation are informative for their own specific health care system because strategies to manage SLE can vary within and between countries. For example, Rydén-Aulin *et al*^11^ estimated that up to 1.2% of patients with SLE in the UK received rituximab whereas up to 4.5% of patients with SLE received rituximab in Sweden. A recent survey of rheumatologists by Keeling *et al*^44^ also reported variation in diagnosing, monitoring and treating SLE within Canada. Structural uncertainty within model-based economic evaluations can occur when different ways of delivering care are possible.^21^ A greater understanding of current practice for SLE can facilitate the development of value propositions for new management strategies; for example, early economic evaluations can be undertaken to identify the key drivers of cost-effectiveness when using precision medicine strategies across the care pathway to stratify treatments or when introducing shared-decision techniques to increase the involvement of patients within their routine prescribing and management decisions. National clinical guidelines, such as the recommendations for SLE published by the British Society for Rheumatology in 2018,^45^ can resolve some structural uncertainty and treatment variation in practice. The use of conceptual models, methods to elicit information formally from experts and further research using observational data from routine clinical decisions could also be used to characterise pathways of care for SLE more appropriately.^46^

The choice of relevant comparators within model-based economic evaluations of SLE can be a challenge due to variations in clinical decisions between jurisdictions. Off-label prescribing or a lack of head-to-head trial evidence are not sufficient grounds to exclude treatments as comparators if they are used as such in routine clinical practice. For example, belimumab was not compared with rituximab (which has been available for a longer period of time) by Specchia *et al*,^32^ yet either biologic agent could be prescribed to the same patient population or used in sequence. The incremental benefit of a health technology can be exaggerated by comparing it with a strategy that is not the next-best alternative; in turn, inappropriate comparators can result in overestimating relative cost-effectiveness.^47^ Greater transparency at the scoping stage of future economic evaluations in SLE may be valuable to define the decision problem and select relevant comparators appropriately.

Innovations in the design of clinical trials for SLE (such as the use of composite end points and inclusion criteria based on molecular biomarkers) have been introduced to address the requirements of regulatory agencies and the limitations of historic trials that failed to reach their primary end points. The requirements of decision-makers that use health technology assessment to inform the reimbursement and recommendation of any new treatment for SLE are different to the requirements of regulatory agencies. Uncertainty in the economic evidence base can inform the need for further collection of data and the design of future primary studies.^48 49^ Only one economic evaluation in this systematic review performed a VOI analysis to investigate the need for further research to reduce uncertainty in the estimates of relative cost-effectiveness. Nee *et al*^30^ estimated the expected value of perfect information (the upper-bound on the cost of future research) to be USD $2058 206; however, estimates of the expected value of partial perfect information, which identify the specific input parameters that would benefit most from future research, were not reported.

The studies identified by this systematic review demonstrated limitations with respect to their estimates of health care resources. Inappropriate estimates of incremental health care resources may have, subsequently, underestimated the opportunity cost (health forgone) of strategies to manage SLE. New techniques to manage SLE (eg, biomarker-based algorithms or shared decision-making tools) will incur additional health care resources that should be quantified appropriately. Microcosting studies may be a valuable source of evidence to estimate the magnitude and value of these additional resources in future model-based economic evaluations in SLE.^50^

The four economic evaluations identified by this systematic review that expressed benefits in quality-adjusted life years had limitations to their estimates of health-related quality of life. Estimates derived from generic preference-based outcomes will likely differ between people with SLE conditional on the specific organ systems involved in their disease. Using a small sample to estimate health-related quality of life for SLE may, therefore, provide values that are not representative of the target population. Disease-specific quality of life measures for SLE comprise domains that are not captured by generic outcome measures explicitly, such as fatigue.^51^ Empirical research to estimate mapping algorithms between disease-specific and generic quality of life measures may be valuable to improve the estimates of health-related quality of life in future model-based economic evaluations in SLE.^52^

National decision-makers provide adoption and research recommendations conditional on subgroup-specific estimates of cost-effectiveness regularly.^53^ People with SLE may comprise subgroups of individuals with different outcomes from treatment across the extent of disease (early diagnosis; treatment selection; treatment monitoring; prediction of flares in disease activity). No study identified by this systematic review reported subgroup-specific estimates of cost-effectiveness based on patient-level variables. Future economic evaluations in SLE could incorporate subgroup-specific estimates of cost-effectiveness or the use of clinical and molecular biomarker information to reveal known heterogeneity in outcomes. Recommendations based on subgroup-specific estimates may, subsequently, lead to improved relative cost-effectiveness and higher population health.^54–56^

One limitation of this systematic review was that only Medline and Embase were searched for published model-based economic evaluations. However, it was unlikely that further studies would have been identified by searching additional databases. A second potential limitation of this study was that the inclusion criteria were restricted to decision-analytic model-based economic evaluations and did not include trial-based designs. Model-based economic evaluations, however, will be more relevant to decisionmakers than trial-based designs because of their ability to extrapolate lifetime cost and health outcomes of people with SLE beyond the duration of short-term RCTs.^57^ In addition, model-based economic evaluations can be designed to inform decisions for a specific target population observed in clinical practice whereas the characteristics of the patients recruited to an RCT may not be representative of this target population.^57^

## Conclusions

Complexity in the diagnosis, management and progression of disease can make model-based economic evaluations of SLE a challenge to undertake. The choice of structural assumptions, characterisation of care pathways and estimation of input parameters can each have an impact on estimates of relative cost-effectiveness. The approaches taken to handle these complexities in the six studies identified by this systematic review have highlighted common challenges faced by authors when producing economic evidence for decisionmakers responsible for population-level health technology assessment. Developments in the methods for decision-analytic model-based cost-effectiveness analysis, such as patient-level simulations, may help analysts to address some of these challenges. New strategies to manage SLE will require economic evidence to support their use in routine clinical practice. The findings from this study can, therefore, be used to improve the quality and relevance of future model-based economic evaluations of treatments for SLE.
