# Supplementary material for: Generating evidence to inform health technology assessment of treatments for SLE: a systematic review of decision-analytic model-based economic evaluations
Source: Lupus Sci Med. 2020 Jul 28;7(1):e000350. doi: 10.1136/lupus-2019-000350 (PMC7389518; doi:10.1136/lupus-2019-000350)
Supplement: Supplementary data [file lupus-2019-000350supp001.pdf]

Generating Evidence to Inform Health Technology Assessment of Treatments for Systemic Lupus Erythematosus: A Systematic Review of Decision-analytic Model-based Economic Evaluations

Supplementary Appendix

Supplementary Material 1: Electronic Search Strategy: Page 2

Supplementary Material 2: Full Data Extraction Tables: Page 4

Gavan S, *et al. Lupus Sci Med* 2020; 7:e000350. doi: 10.1136/lupus-2019-000350

Supplementary Appendix 1: Electronic Search Strategy

Medline

1 Economics/
2 exp "costs and cost analysis"/
3 Economics, Dental/
4 exp economics, hospital/
5 Economics, Medical/
6 Economics, Nursing/
7 Economics, Pharmaceutical/
8 (economic$ or cost or costs or costly or costing or price or prices or pricing or pharmacoeconomic$).ti,ab.
9 (expenditure$ not energy).ti,ab.
10 (value adj1 money).ti,ab.
11 budget$.ti,ab.
12 or/1-11
13 ((energy or oxygen) adj cost).ti,ab.
14 (metabolic adj cost).ti,ab.
15 ((energy or oxygen) adj expenditure).ti,ab.
16 or/13-15
17 12 not 16
18 exp animals/ not humans/
19 17 not 18
20 Lupus Erythematosus, Systemic/
21 Lupus Nephritis/
22 Lupus Erythematosus, Cutaneous/
23 or/20-22
24 (Systemic lupus erythematosus* or Systemic-lupus-erythematosus* or SLE or lupus* or nephritis).mp.
25 23 or 24
26 19 and 25
27 remove duplicates from 26

Embase

1 health-economics/
2 exp economic-evaluation/
3 exp health-care-cost/
4 exp pharmacoeconomics/
5 1 or 2 or 3 or 4
6 (econom$ or cost or costs or costly or costing or price or prices or pricing or pharmacoeconomic$).ti,ab.
7 (expenditure$ not energy).ti,ab.
8 (value adj2 money).ti,ab.
9 budget$.ti,ab.
10 6 or 7 or 8 or 9
11 5 or 10
12 (metabolic adj cost).ti,ab.
13 ((energy or oxygen) adj cost).ti,ab.
14 ((energy or oxygen) adj expenditure).ti,ab.
15 12 or 13 or 14
16 11 not 15
17 animal/
18 exp animal experiment/
19 nonhuman/
20 (rat or rats or mouse or mice or hamster or hamsters or animal or animals or dog or dogs or cat or cats or bovine or sheep).ti,ab,sh.
21 17 or 18 or 19 or 20
22 16 not 21
23 exp systemic lupus erythematosus/
24 exp lupus erythematosus nephritis/
25 exp skin lupus erythematosus/
26 (Systemic lupus erythematosus* or Systemic-lupus-erythematosus* or SLE or lupus* or nephritis).ti,ab.
27 23 or 24 or 25 or 26
28 22 and 27
29 conference abstract.pt.
30 28 not 29
31 remove duplicates from 30

Gavan S, *et al. Lupus Sci Med* 2020; 7:e000350. doi: 10.1136/lupus-2019-000350

Supplementary Appendix 2: Full Data Extraction Tables

Table S1. Full Data Extraction Table for Marra et al. (2002) [1].

Study Design	Study Characteristics	Data Sources	Analysis	Results
**Target population**:<br>Patients with rheumatological conditions (predominantly RA and SLE).<br><br>**Alternatives**:<br>(i) Usual full dose azathioprine;<br>(ii) PCR genotype test of TPMT activity to inform dose of azathioprine.<br><br>**Country**:<br>Canada.	**Evaluation method**:<br>CEA.<br><br>**Model type**:<br>Decision tree.<br><br>**Time horizon**:<br>One year.<br><br>**Perspective**:<br>Third party payer.<br><br>**Benefit measure**:<br>Adverse drug reactions avoided.<br><br>**Direct costs included**:<br>PCR testing; Treatments; Routine laboratory tests; Physician office visits.<br><br>**Indirect costs included**:<br>Not applicable.	**Effectiveness**:<br>Adverse events from azathioprine [2]; Effectiveness of testing based on an assumption; Test accuracy [3].<br><br>**Health-related quality of life**:<br>Not applicable.<br><br>**Resource use**:<br>Probability and duration of hospitalisations estimated by experts; Number of visits based on an assumption.<br><br>**Unit cost**s:<br>Cost of testing by proxy with other PCR tests; Hospital cost model [4]; Provincial Guide to Medical Fees; IMS Health Canada Database.<br><br>**Discount rate**:<br>Not applicable.<br><br>**Currency (Price year)**:<br>Canadian $ (1999).	**Deterministic sensitivity**:<br>Probability of preventable adverse drug reactions; Cost of PCR testing; Probability of hospitalisation; Test accuracy.<br><br>**Probabilistic sensitivity**:<br>No.<br><br>**Value of information**:<br>No.	**Base-case**:<br>Genotype test to inform azathioprine dose was dominant.<br><br>**Probabilistic analysis**:<br>Not applicable.<br><br>**Value of information**:<br>Not applicable.<br><br>**Key drivers of relative cost-effectiveness**:<br>Cost of PCR testing; Probability of hospitalisations.

Abbreviations: CEA: Cost-effectiveness analysis; PCR: Polymerase chain reaction; RA: Rheumatoid arthritis; SLE: Systemic lupus erythematosus; TPMT: Thiopurine S-methyltransferase.

Table S2. Full Data Extraction Table for Mohara et al. (2014) [5].

Study Design	Study Characteristics	Data Sources	Analysis	Results
**Target population**:<br>Patients, aged 40, newly diagnosed with active, severe lupus nephritis and receiving immunosuppressive therapy.<br><br>**Alternatives**:<br>(i) IV cyclophosphamide (1,000mg/m^2 per month) for 6 months induction and IV cyclophosphamide every 3 months for 3 years maintenance;<br>(ii) IV cyclophosphamide (1,000mg/m^2 per month) for 6 months induction and azathioprine (50mg per day) for 3 years maintenance;<br>(iii) IV cyclophosphamide (1,000mg/m^2 per month) for 6 months induction and MMF (1000mg per day) for 3 years maintenance;<br>(iv) MMF (2,000mg per day) for 6 months induction, then MMF (1,000mg per day) for 6 months, then azathioprine (50mg per day) as maintenance for up to 3 years.<br><br>**Country**:<br>Thailand.	**Evaluation method**:<br>CUA.<br><br>**Model type**:<br>Markov model.<br><br>**Time horizon**:<br>Lifetime.<br><br>**Perspective**:<br>Societal.<br><br>**Benefit measure**:<br>QALYs.<br><br>**Direct costs included**:<br>Treatments; Laboratory tests; Administrative costs.<br><br>**Indirect costs included**:<br>Transportation; Meals; Informal Care; Accommodations; Facilities; Productivity loss.	**Effectiveness**:<br>Transition probabilities [6-8]<br>Relative treatment effects: Induction phase [9]; Maintenance phase from accompanying meta-analysis.<br><br>**Health-related quality of life**:<br>Direct observation (18 patients) in 4 Thai hospitals.<br><br>**Resource use**:<br>Medical records in 4 Thai hospitals.<br><br>**Unit cost**s:<br>Thailand Ministry of Public Health; Standard Cost List for Health Technology Assessment; Thailand Hospital Database; ESRD [10].<br><br>**Discount rate**:<br>3% for costs and health.<br><br>**Currency (Price year)**:<br>Thai baht (2012)	**Deterministic sensitivity**:<br>One-way sensitivity analysis on all input parameters.<br><br>**Probabilistic sensitivity**:<br>Yes.<br><br>**Value of information**:<br>No.	**Base-case**:<br>Strategy (ii) was dominant.<br><br>**Probabilistic analysis**:<br>Strategy (ii) had the highest probability of cost-effectiveness across a range of cost-effectiveness thresholds.<br><br>**Value of information**:<br>Not applicable.<br><br>**Key drivers of relative cost-effectiveness**:<br>Relative risk of complete remission, partial remission, and renal failure.

Abbreviations: CUA: Cost-utility analysis; ESRD: End-stage renal disease; IV: Intravenous; MMF: Mycophenolate mofetil; QALY: Quality-adjusted life year.

Table S3. Full Data Extraction Table for Nee et al. (2015) [11].

Study Design	Study Characteristics	Data Sources	Analysis	Results
**Target population**:<br>Patients with lupus nephritis, between 20 and 40 years, that had responded to their induction therapy.<br><br>**Alternatives**:<br>(i) Azathioprine (150mg per day);<br>(ii) MMF (2mg per day).<br><br>**Country**:<br>United States of America.	**Evaluation method**:<br>CUA.<br><br>**Model type**:<br>Markov microsimulation model.<br><br>**Time horizon**:<br>3 years and lifetime.<br><br>**Perspective**:<br>Societal.<br><br>**Benefit measure**:<br>QALYs.<br><br>**Direct costs included**:<br>Treatments, patient visits, laboratory studies, imaging studies, emergency visits, outpatient surgery, hospitalisations.<br><br>**Indirect costs included**:<br>Patient labour and non-labour time, caregiver time delivering care.	**Effectiveness**:<br>Transition probabilities [12-15].<br><br>**Health-related quality of life**:<br>Published values derived from VAS and TTO [16-19].<br><br>**Resource use**:<br>The Red Book, Tri-nation cohort [16, 20, 21].<br><br>**Unit cost**s:<br>The Red Book, Tri-nation cohort [16, 20, 21].<br><br>**Discount rate**:<br>3% for costs and health.<br><br>**Currency (Price year)**:<br>$, USA (2013).	**Deterministic sensitivity**:<br>Single trial for treatment effectiveness; indirect costs in remission; utility in remission; cost of treatments; probability of infection; probability of end stage renal disease; direct costs during remission; disutility from infection; probability of relapse.<br><br>**Probabilistic sensitivity**:<br>Yes.<br><br>**Value of information**:<br>Population expected value of perfect information for the three-year model.	**Base-case**:<br>In the lifetime model, MMF had an ICER of $6,454 per QALY gained, relative to azathioprine.<br><br>**Probabilistic analysis**:<br>The probability that MMF was cost-effective was close to 100% for thresholds of $50,000 and $100,000 per QALY gained.<br><br>**Value of information**:<br>Population EVPI was $2,058,206 at a threshold of $100,000 per QALY gained for the three-year model.<br><br>**Key drivers of relative cost-effectiveness**:<br>For the three-year model: indirect costs during remission; utility in remission; cost of azathioprine.

Abbreviations: CUA: Cost-utility analysis; EVPI: Expected value of perfect information; ICER: Incremental cost-effectiveness ratio; MMF: Mycophenolate mofetil; QALY: Quality-adjusted life year; TTO: Time trade-off; VAS: Visual analogue scale.

Gavan S, *et al. Lupus Sci Med* 2020; 7:e000350. doi: 10.1136/lupus-2019-000350

Table S4. Full Data Extraction Table for Oh et al. (2004) [22].

Study Design	Study Characteristics	Data Sources	Analysis	Results
**Target population**:<br>Adults (50kg body weight) with moderate to severe rheumatoid arthritis or SLE who are unsuitable for methotrexate or cyclophosphamide.<br><br>**Alternatives**:<br>(i) Conventional weight-based dose of azathioprine;<br>(ii) PCR genotype test of TPMT activity to inform dose of azathioprine.<br><br>**Country**:<br>Korea.	**Evaluation method**:<br>CEA.<br><br>**Model type**:<br>Decision tree.<br><br>**Time horizon**:<br>One year.<br><br>**Perspective**:<br>Societal.<br><br>**Benefit measure**:<br>Azathioprine discontinuation due to severe adverse events.<br><br>**Direct costs included**:<br>Treatments, routine laboratory tests, PCR genotype test, hospital admissions.<br><br>**Indirect costs included**:<br>Not reported.	**Effectiveness**:<br>Prevalence of heterogeneity in TPMT activity [23]; Test accuracy [3]; Incidence of severe adverse events [24].<br><br>**Health-related quality of life**:<br>Not applicable.<br><br>**Resource use**:<br>Treatment guidelines from the Physician's Desk Reference, Hospitalisations from four cases observed at Hanyang University Hospital.<br><br>**Unit cost**s:<br>Korean insurance system, Four cases observed at Hanyang University Hospital.<br><br>**Discount rate**:<br>Not reported.<br><br>**Currency (Price year)**:<br>Korean won (2002).	**Deterministic sensitivity**:<br>Prevalence of TPMT activity, Cost of hospital admission, Cost of PCR genotype test, Incidence of severe adverse events.<br><br>**Probabilistic sensitivity**:<br>No.<br><br>**Value of information**:<br>No.	**Base-case**:<br>Genotype test to inform azathioprine dose was dominant.<br><br>**Probabilistic analysis**:<br>Not applicable.<br><br>**Value of information**:<br>Not applicable.<br><br>**Key drivers of relative cost-effectiveness**:<br>Result was not sensitive to changes in input parameters.

Abbreviations: CEA: Cost-effectiveness analysis; PCR: Polymerase chain reaction; SLE: Systemic lupus erythematosus; TPMT: Thiopurine S-methyltransferase.

Gavan S, *et al. Lupus Sci Med* 2020; 7:e000350. doi: 10.1136/lupus-2019-000350

Table S5. Full Data Extraction Table for Specchia et al. (2014) [25].

Study Design	Study Characteristics	Data Sources	Analysis	Results
**Target population**:<br>50,000 patients with SLE that had active disease and a positive autoantibody test (anti-dsDNA positive and low complement).<br><br>**Alternatives**:<br>(i) Belimumab (10mg per kilogram) plus standard of care;<br>(ii) Standard of care alone (corticosteroids, antimalarial agents, NSAIDs, cytotoxic chemotherapy, immunosuppressive and immunomodulatory drugs).<br><br>**Country**:<br>Italy.	**Evaluation method**:<br>CEA; CUA.<br><br>**Model type**:<br>Individual-level microsimulation.<br><br>**Time horizon**:<br>Lifetime.<br><br>**Perspective**:<br>Italian National Health Service and Societal.<br><br>**Benefit measure**:<br>Life years; QALYs.<br><br>**Direct costs included**:<br>Diagnostic tests; specialist visits; organ damage.<br><br>**Indirect costs included**:<br>Not reported explicitly.	**Effectiveness**:<br>Accompanying systematic review of the literature; BLISS trials [26] and the Johns Hopkins observational cohort [27].<br><br>**Health-related quality of life**:<br>Published literature, based on the BLISS trial sample of UK patients.<br><br>**Resource use**:<br>Not reported.<br><br>**Unit cost**s:<br>Published national tariff and international literature [28-30]. Human capital approach for indirect costs.<br><br>**Discount rate**:<br>3% for costs and health.<br><br>**Currency (Price year)**:<br>Euro (2011).	**Deterministic sensitivity**:<br>Change in SELENA-SLEDAI at 1-year; change in SELENA-SLEDAI according to the natural history model; discontinuation rate; probability of response; mortality and organ damage probabilities; mortality rates; utility; organ damage disutility; costs of each SELENA-SLEDAI score; organ damage cost; indirect costs.<br><br>**Probabilistic sensitivity**:<br>Yes.<br><br>**Value of information**:<br>No.	**Base-case**:<br>For NHS perspective, belimumab had an ICER of €32,859 per QALY gained, and €22,990 per life year gained, compared with the standard of care.<br><br>**Probabilistic analysis**:<br>At a threshold of €30,000 per QALY gained, the probability that belimumab was cost-effective was 29.1%. At €40,000 per QALY gained, the probability was 84.3%.<br><br>**Value of information**:<br>Not applicable.<br><br>**Key drivers of relative cost-effectiveness**:<br>Treatment effect; discontinuation rate.

Abbreviations: BLISS: Belimumab in Subjects with SLE; CEA: Cost-effectiveness analysis; CUA: Cost-utility analysis; ICER: Incremental cost-effectiveness ratio; NHS: National Health Service; NSAID: Non-steroidal anti-inflammatory drug; QALY: Quality-adjusted life year; SELENA-SLEDAI: Safety of Estrogens in Lupus National Assessment-Systemic Lupus Erythematosus Disease Activity Index; SLE: Systemic lupus erythematosus.

Gavan S, *et al. Lupus Sci Med* 2020; 7:e000350. doi: 10.1136/lupus-2019-000350

Table S6. Full Data Extraction Table for Wilson et al. (2007) [31].

Study Design	Study Characteristics	Data Sources	Analysis	Results
**Target population**:<br>10,000 patients with lupus nephritis that were due to receive induction therapy for a flare.<br><br>**Alternatives**:<br>(i) MMF plus prednisolone;<br>(ii) IV cyclophosphamide plus prednisolone.<br><br>**Country**:<br>United Kingdom.	**Evaluation method**:<br>CUA.<br><br>**Model type**:<br>Patient-level simulation.<br><br>**Time horizon**:<br>6 months.<br><br>**Perspective**:<br>UK NHS.<br><br>**Benefit measure**:<br>QALYs.<br><br>**Direct costs included**:<br>Treatments and administration; major and minor infections.<br><br>**Indirect costs included**:<br>Not applicable.	**Effectiveness**:<br>Published systematic review [32] of RCTs [7, 33]; Published Cochrane review [34].<br><br>**Health-related quality of life**:<br>Published sources of disutility associated with infection [35-37].<br><br>**Resource use**:<br>British National Formulary; Published sources.<br><br>**Unit costs**:<br>British National Formulary; Department of Health Reference Costs; Unit Costs of Health and Social Care.<br><br>**Discount rate**:<br>Not applicable.<br><br>**Currency (Price year)**:<br>£ sterling (2005).	**Deterministic sensitivity**:<br>Response rate to MMF and IV cyclophosphamide; rate of major and minor infections from both alternatives; duration of major and minor infections; rate of switching between alternatives; dose of treatments; disutility associated with each level of response; disutility associated with major and minor infections.<br><br>**Probabilistic sensitivity**:<br>Yes.<br><br>**Value of information**:<br>No.	**Base-case**:<br>The base-case analysis indicated that MMF dominated IV cyclophosphamide.<br><br>**Probabilistic analysis**:<br>At a cost-effectiveness threshold of between £25,000 to £35,000 per QALY gained, MMF had an 81% probability of being cost-effective.<br><br>**Value of information**:<br>Not applicable.<br><br>**Key drivers of relative cost-effectiveness**:<br>The response rate to IV cyclophosphamide.

Abbreviations: CUA: Cost-utility analysis; IV: Intravenous; MMF: Mycophenolate mofetil; NHS: National Health Service; QALY: Quality-adjusted life year; RCT: Randomised controlled trial.

Supplementary Appendix References

1. Marra C, Esdaile J, Anis A. Practical Pharmacogenetics: The Cost Effectiveness of Screening for Thiopurine S-methyltransferase Polymorphisms in Patients with Rheumatological Conditions Treated with Azathioprine. J Rheumatol. 2002;29(12):2507-2512.

2. Suarez-Almazor M, Spooner C, Belseck E. Azathioprine for Treating Rheumatoid Arthritis (Review). Cochrane Database Syst Rev. 2000;4(CD00146):1-17.

3. Yates C, Krynetski E, Loennechen T, et al. Molecular Diagnosis of Thiopurine S-methyltransferase Deficiency: Genetic Basis for Azathioprine and Mercaptopurine Intolerance. Ann Intern Med. 1997;126(8):608-614.

4. Anis A, Guh D, Stieb D, et al. The Costs of Cardiorespiratory Disease Episodes in a Study of Emergency Department use. Can J Public Health. 2000;91(2):103-106.

5. Mohara A, Pérez Velasco R, Praditsitthikorn N, et al. A Cost-utility Analysis of Alternative Drug Regimens for Newly Diagnosed Severe Lupus Nephritis Patients in Thailand. Rheumatology. 2014;53(1):138-144.

6. de Melo A, Avelar A, Maegawa F, et al. Analysis of 100 Patients with Lupus Nephritis Followed up for 2 Years. Revista Brasileira de Reumatologia. 2009;49(1):8-19.

7. Ong L, Hooi L, Lim T, et al. Randomized Controlled Trial of Pulse Intravenous Cyclophosphamide Versus Mycophenolate Mofetil in the Induction Therapy of Proliferative Lupus Nephritis. Nephrology. 2005;10(5):504-510.

8. Illei G, Takada K, Parkin D, et al. Renal Flares are Common in Patients with Severe Proliferative Lupus Nephritis Treated with Pulse Immunosuppressive Therapy: Long-term Followup of a Cohort of 145 Patients Participating in Randomized Controlled Studies. Arthritis Rheum. 2002;46(4):995-1002.

9. Lee Y, Woo J, Choi S, et al. Induction and Maintenance Therapy for Lupus Nephritis: A Systematic Review and Meta-analysis. Lupus. 2010;19(6):703-710.

10. Teerawattananon Y, Mugford M, Tangcharoensathien V. Economic Evaluation of Palliative Management Versus Peritoneal Dialysis and Hemodialysis for End-stage Renal Disease: Evidence for Coverage Decisions in Thailand. Value Health. 2007;10(1):61-72.

11. Nee R, Rivera I, Little D, et al. Cost-Utility Analysis of Mycophenolate Mofetil versus Azathioprine Based Regimens for Maintenance Therapy of Proliferative Lupus Nephritis. International Journal of Nephrology. 2015;2015(917567):1-13.

12. Henderson L, Masson P, Craig J, et al. Treatment for Lupus Nephritis (Review). Cochrane Database Syst Rev. 2012;12(CD002922).

13. Costenbader K, Desai A, Alarcón G, et al. Trends in the Incidence, Demographics, and Outcomes of End-stage Renal Disease due to Lupus Nephritis in the US from 1995 to 2006. Arthritis Rheum. 2011;63(6):1681-1688.

14. Bernatsky S, Boivin J, Joseph L, et al. Mortality in Systemic Lupus Erythematosus. Arthritis Rheum. 2006;54(8):2550-2557.

15. Sule S, Fivush B, Neu A, et al. Increased Risk of Death in Pediatric and Adult Patients with ESRD Secondary to Lupus. Pediatr Nephrol. 2011;26(1):93-98.

16. Clarke A, Panopalis P, Petri M, et al. SLE Patients with Renal Damage Incur Higher Health Care Costs. Rheumatology. 2008;47(3):329-333.

Gavan S, *et al. Lupus Sci Med* 2020; 7:e000350. doi: 10.1136/lupus-2019-000350

17. Liem Y, Bosch J, Hnink M. Preference-based Quality of Life of Patients on Renal Replacement Therapy: A Systematic Review and Meta-analysis. Value Health. 2008;11(4):733-741.

18. Grootscholten C, Snoek F, Bijl M, et al. Health-related Quality of Life and Treatment Burden in Patients with Proliferative Lupus Nephritis Treated with Cyclophosphamide or Azathioprine/Methylprednisolone in a Randomized Controlled Trial. J Rheumatol. 2007;34(8):1699-1707.

19. Tse K, Tang C, Lio W, et al. Quality of Life Comparison between Corticosteroid- and-Mycofenolate Mofetil and Corticosteroid- and-oral Cyclophosphamide in the Treatment of Severe Lupus Nephritis. Lupus. 2006;15(6):371-379.

20. Clarke A, Petri M, Manzi S, et al. The Systemic Lupus Erythematosus Tri-nation Study: Absence of a Link between Health Resource use and Health Outcome. Rheumatology. 2004;43(8):1016-1024.

21. Panopalis P, Petri M, Manzi S, et al. The Systemic Lupus Erythematosus Tri-Nation Study: Cumulative Indirect Costs. Arthritis Rheum. 2007;57(1):64-70.

22. Oh K, Anis A, Bae S. Pharmacoeconomic Analysis of Thiopurine Methyltransferase Polymorphism Screening by Polymerase Chain Reaction for Treatment with Azathioprine in Korea. Rheumatology. 2004;43(2):156-163.

23. Weinshilboum R. Methyltransferase Pharmacogenetics. Pharmacol Ther. 1989;43(1):77-90.

24. Connell W, Kamm M, Ritchie J, et al. Bone Marrow Toxicity Caused by Azathioprine in Inflammatory Bowel Disease: 27 Years of Experience. Gut. 1993;34(8):1081-1085.

25. Specchia M, de Waure C, Gualano M, et al. Health Technology Assessment of Belimumab: A New Monoclonal Antibody for the Treatment of Systemic Lupus Erythematosus. BioMed Research International. 2014;2014(704207):1-9.

26. Petri M, Levy R, Merrill J, et al. Belimumab, a BLyS-Specific Inhibitor, Reduced Disease Activity, Flares, and Prednisone Use in Patients with Seropositive SLE: Combined Efficacy Results from the Phase 3 BLISS-52 and -76 Studies. Arthritis Rheum. 2010;62(S10):S190.

27. Petri M. Lupus in Baltimore: Evidence-based 'Clinical Pearls' from the Hopkins Lupus Cohort. Lupus. 2005;14(12):970-973.

28. Fattore G, Torbica A. Cost and Reimbursement of Cataract Surgery in Europe: A Cross-country Comparison. Health Econ. 2008;17(S1):S71-S82.

29. Pugliatti M, Sobocki P, Beghi E, et al. Cost of Disorders of the Brain in Italy. Neurol Sci. 2008;29(2):99-107.

30. Piscitelli P, Iolascon G, Greco M, et al. The Occurrence of Acute Myocardial Infarction in Italy: A Five-year Analysis of Hospital Discharge Records. Aging Clin Exp Res. 2011;23(1):49-54.

31. Wilson E, Jayne D, Dellow E, et al. The Cost-effectiveness of Mycophenolate Mofetil as Firstline Therapy in Active Lupus Nephritis. Rheumatology. 2007;46(7):1096-1101.

32. Moore R, Derry S. Systematic Review and Meta-analysis of Randomised Trials and Cohort Studies of Mycophenolate Mofetil in Lupus Nephritis. Arthritis Res Ther. 2006;8(R128):1-10.

33. Ginzler E, Dooley M, Aranow C, et al. Mycophenolate Mofetil or Intravenous Cyclophosphamide for Lupus Nephritis. N Engl J Med. 2005;353(21):2219-2228.

34. Flanc R, Roberts M, Strippoli G, et al. Treatment for Lupus Nephritis. Cochrane Database Syst Rev. 2004;1(CD002922):1-89.

35. Balk E, Zucker D, Engels E, et al. Strategies for Diagnosing and Treating Suspected Acute Bacterial Sinusitis: A Cost-effectiveness Analysis. J Gen Intern Med. 2001;16(10):701-711.

36. Smith K, Roberts M. Antiviral Therapies for Herpes Zoster Infections. Are they Economically Justifiable? Pharmacoeconomics. 2000;18(2):95-104.

37. Smith K, Roberts M. Cost Effectiveness of Newer Antiviral Agents for Herpes Zoster: Is the Evidence Spotty? J Infect Dis. 1998;178(S1):S85-S90.

Gavan S, *et al. Lupus Sci Med* 2020; 7:e000350. doi: 10.1136/lupus-2019-000350
